# Aneurism after Venesection Cured by Flexion of the Limb

**Published:** 1853-02

**Authors:** 


*Aneurism after Venesection cured by flexion of the Limb.*—M. A. Thierry has lately published, in the *Revue Clinique,* a case of false aneurism at the bend of the elbow, occurring after bleeding from the arm, which he successfully treated in the following manner:—The arm was forcibly flexed, the limb carried over the head, and the hand fixed on the opposite cheek. The patient remained in this painful position for five days, after which time it was changed to that which M. Velpeau generally adopts for fracture of the clavicle—viz., the arm fixed across the chest, and the opposite shoulder. A fortnight after the beginning of this treatment, the tumor was reduced to the size of a nut; the arm was then kept in the same position for another fortnight, after which no sign of any pulsating tumor remained. M. Nélaton, who saw the patient, considered the case a very remarkable one, as the aneurism has disappeared, and the vessel remains permeable at the seat of the wound. M. Thierry very justly says, that one case is not sufficient to prove the efficacy of any method of treatment, but that the results here obtained are well worthy of attention; he thinks that further trials will perhaps lead surgeons to treat aneurisms of the limbs by forced flexion, femoral aneurism by flexion of the thigh upon the pelvis, and popliteal aneurism by flexing that leg upon the thigh. If we mistake not, M. Thierry's method is founded upon the principle of pressure, and carried out with a great deal of pain and inconvenience to the patient. If the flow of arterial blood through the sac can be graduated, moderated, and rendered very slow by simple and painless means (as is proved by experience,) it is cruel to torture patients by placing them for a whole month in the position given by the immortal statuary to Laocoon.—*Lancet.*

